# The Evolving Clinical Trajectory From a Sliding Hiatus Hernia to Mixed Sliding and Para-Oesophageal Subtype: A Case Report

**DOI:** 10.7759/cureus.56287

**Published:** 2024-03-16

**Authors:** Aimee Hughes, Muhammad Ibrahim Shahzad, Mansoor Zafar, Khalil El Gendy, Julian R.F. Walters

**Affiliations:** 1 Gastroenterology, Hammersmith Hospital - Imperial College Healthcare NHS Trust, London, GBR; 2 Radiology, Hammersmith Hospital - Imperial College Healthcare NHS Trust, London, GBR

**Keywords:** intra-gastric reflux, barium swallow, hill classification, rolling para oesophageal hiatus hernia, sliding hiatus hernia

## Abstract

We present a compelling case of a patient initially diagnosed with a simple sliding hiatus hernia (HH), which was managed conservatively through optimised medical therapy. Over the span of a few years, she developed new symptoms which included epigastric discomfort and pain, prompting further clinical review and imaging investigation. These revealed the progression of her HH from a simple form to a more complex rolling or para-oesophageal type. This outcome highlights the importance of recognising a potential for progression during the clinical assessment of patients with a history of reflux symptoms and the onset of new epigastric discomfort or pain. Understanding this continuum of HHs is essential for physicians as management plans may need to switch from a conservative to a more invasive approach.

## Introduction

Hiatus hernias (HHs) have been reported to affect around 1-20% of the population although only up to 9% have been further reported to be symptomatic. Up to 95% of symptomatic patients seem to have a sliding HH involving propulsion of only the lower oesophageal sphincter into the chest through the diaphragmatic crura. About 5% of symptomatic cases have a para-oesophageal or rolling HH where the lower oesophageal sphincter (LOS) remains below the diaphragm, and it is the proximal portion of the stomach fundus that pushes above the diaphragm [1]. The prevalence has been reported to increase with advancing age [1,2]. The muscular diaphragmatic aperture allowing the oesophagus to pass distally to communicate with the stomach is labelled as the gastro-oesophageal junction (GOJ). In an HH, the LOS is compromised and the consequential backup of gastric contents into the oesophagus leads to the sensation of acid reflux known as gastro-oesophageal reflux disease (GORD) [2] and epigastric discomfort or pain. This stems from the upper part of the stomach not being able to return to its natural position below the diaphragm during swallowing. A contributing factor is increased intra-abdominal pressure associated with obesity, chronic constipation, chronic obstructive pulmonary disease and pregnancy. Other reported causes include trauma, advancing age, a history of surgeries in the past and genetics [3]. Interestingly, HHs have been reported to be more prevalent in Western Europe and North America and comparatively a rarer occurrence in rural Africa [4]. 

We report an interesting case where over time a simple sliding HH progressed to a mixed sliding HH along with a rolling para-oesophageal HH.

## Case presentation

A 53-year-old lady was reviewed in the gastroenterology clinic with persistent reflux symptoms, ongoing for almost five years despite the use of proton pump inhibitors (PPIs). Her background included undifferentiated inflammatory arthritis with positive anti-CCP and mild arthralgia symptoms affecting her hands, knees and ankles (HLA-B27 negative). She also had stable Crohn’s disease, fibromyalgia, heterozygous familial hypercholesterolemia, a history of unilateral transient ischaemic attack with limb weakness and speech impairment that resolved following a left-sided carotid endarterectomy a year ago. She also had a history of hypothyroidism, asthma, anxiety, and depression. She quit smoking 16 years ago, before which she was a heavy smoker and was also being investigated by the respiratory team for interstitial lung disease and had periodic computed tomograms (CTs) of the chest.

**Figure 1:**
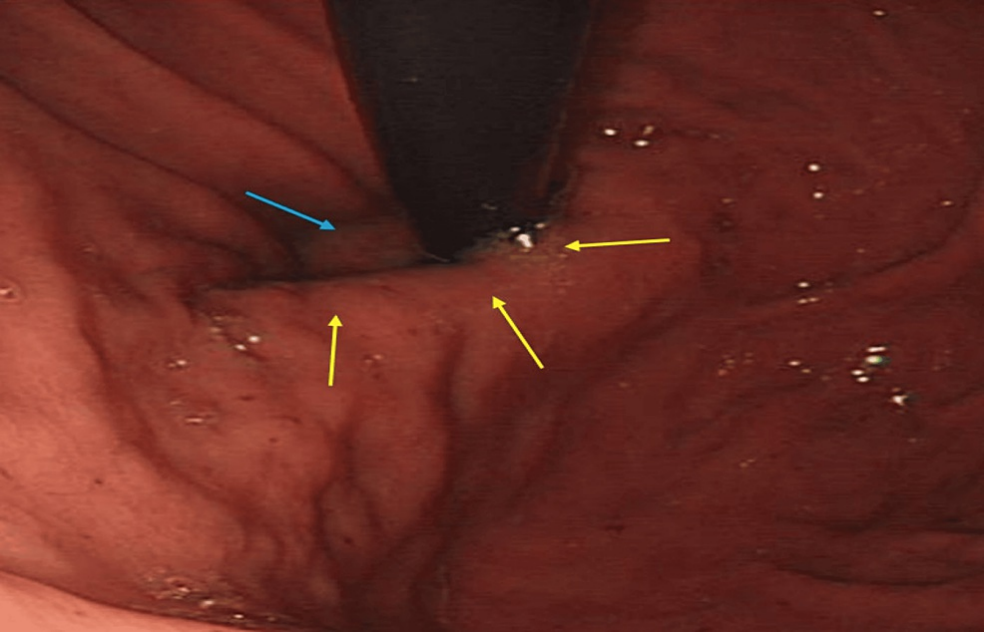
Sliding hiatus hernia (yellow arrows), with lax-lower GOJ junction (blue arrow) on anticlockwise rotation of the endoscope with retroflexion of stomach fundus. GOJ: Gastro-oesophageal junction

On further inquiry, she described a sensation of food being stuck which had been ongoing for the last six months with occasional epigastric pain. Her weight was stable and her appetite was reasonable. Additionally, she noted an acid-brash sensation in her mouth in the early hours of the morning and late at night. Her previous endoscopy two years before showed evidence of a sliding HH (Figure 1).

**Figure 2:**
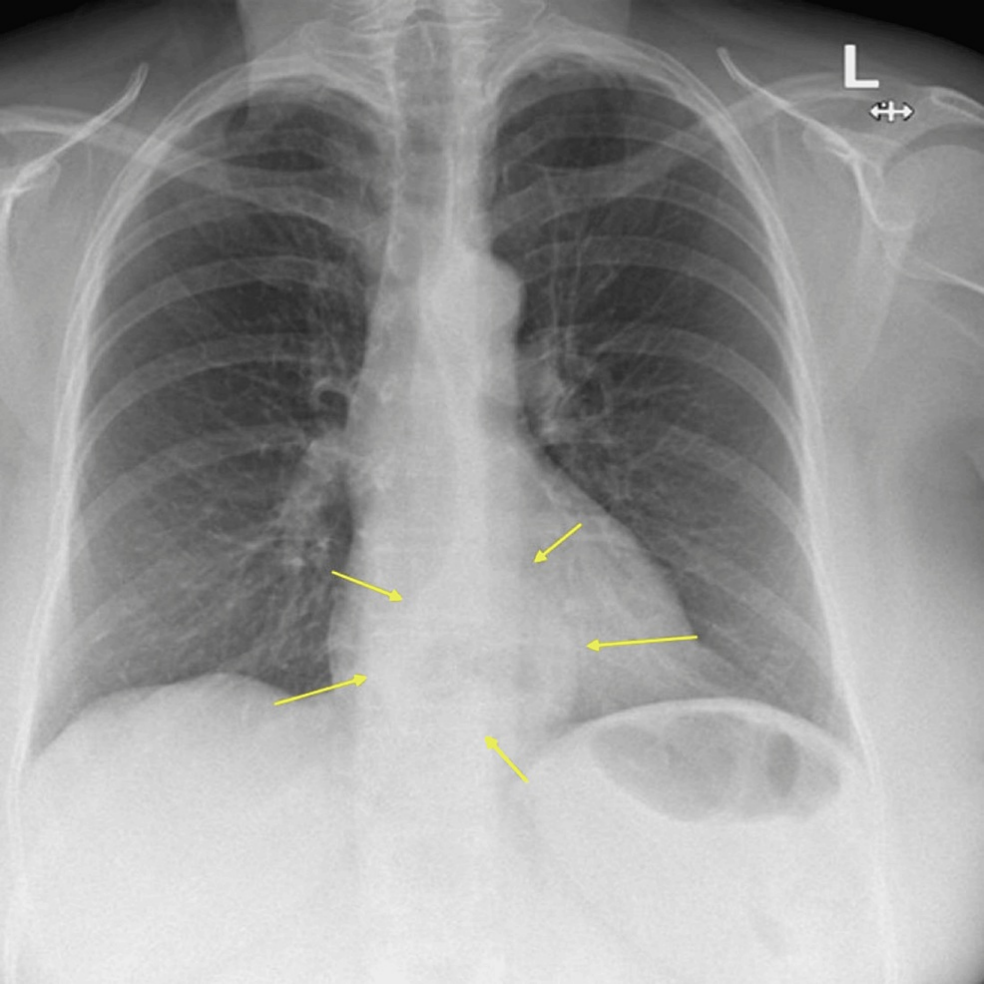
Chest X-ray. Posterior-anterior (PA) view with hyperinflated lung fields with a sliding hiatus hernia (yellow arrow).

She also had a chest X-ray two years before, arranged due to a lower respiratory tract infection, and this supported the finding of a sliding HH (Figure 2). 

**Figure 3:**
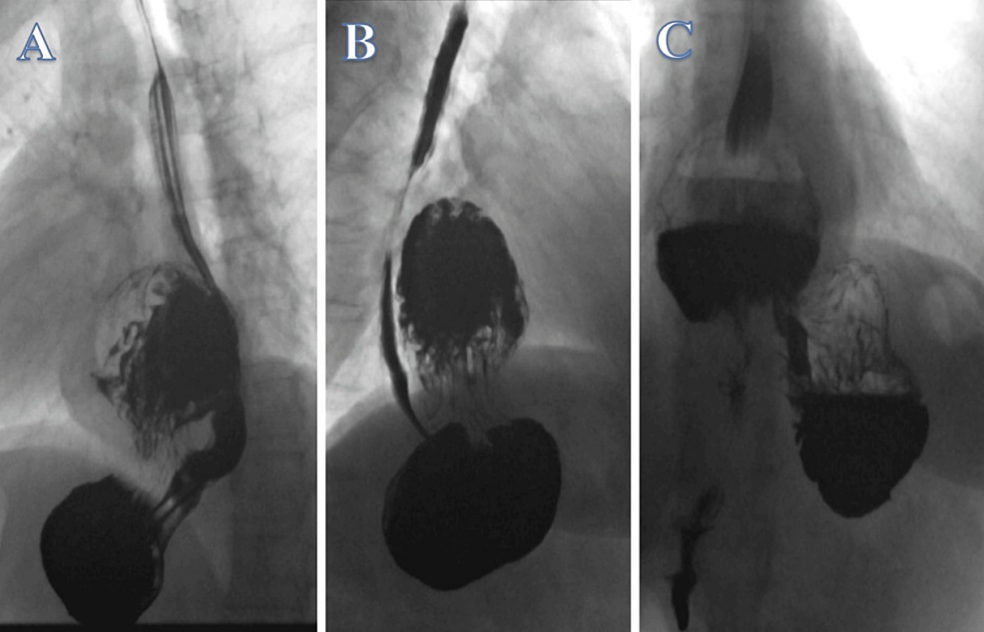
Barium swallow. Contrast passing smoothly to the fundus and proximal part (A) and then to the herniated part (B). Retrograde stream into the proximal stomach in keeping with intragastric reflux (C).

The patient was referred for barium swallow studies. These showed findings of a moderate-sized rolling para-oesophageal HH, containing part of the gastric body. The contrast seems to pass smoothly initially to the fundus and proximal part and then to the herniated part. Following this event, the contrast seems to retrograde into the proximal stomach keeping with intra-gastric reflux (Figure 3, Video 1).

**Video 1:** Barium swallow. Contrast passing smoothly to the fundus and proximal part and then to the herniated part. Following notice the retrograde stream into the proximal stomach in keeping with intragastric reflux.

**Figure 4:**
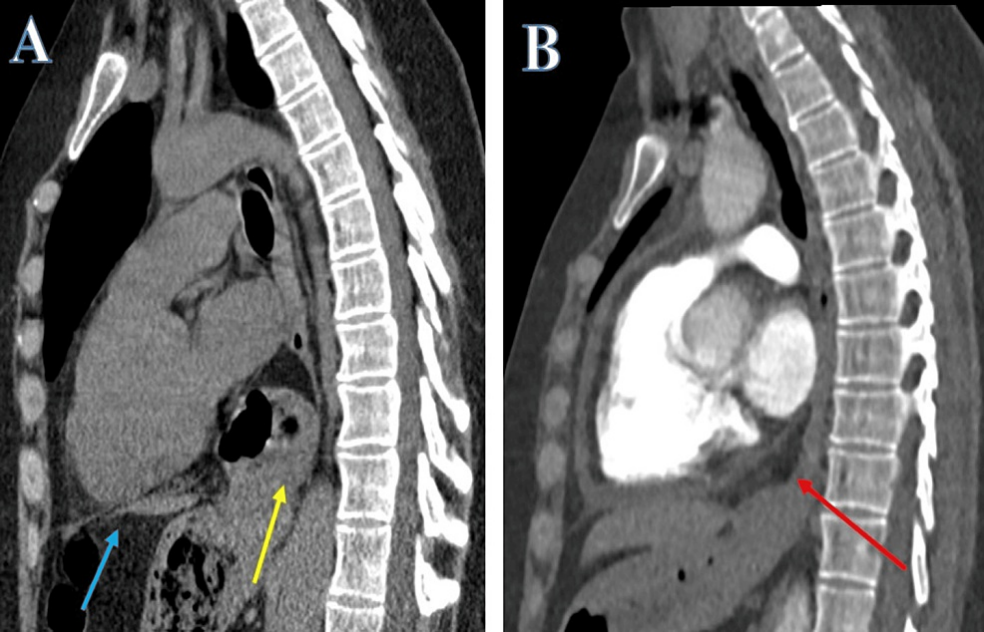
Computed tomogram- sagittal view. Progression of the HH. Sliding hiatus hernia five years ago (yellow arrow) above the diaphragm (blue arrow) (A) that seems to have progressed to a rolling para-oesophageal hiatus hernia (red arrow) (B) in a recent CT chest scan.

The CT scan surveillance, performed to rule out any lung involvement with the positive anti-CCP undifferentiated inflammatory arthritis and as part of autoimmune disease workup to rule out pulmonary involvement, confirmed the transition from a sliding to a rolling HH (Figure 4).

The patient was instructed to maintain their regimen of a PPI (omeprazole 40 mg twice daily) and was referred to the upper gastrointestinal (GI) surgical team for potential surgical intervention.

This intriguing case highlights the evolving clinical trajectory of the sliding HH, initially detected on endoscopy and chest X-ray, which appears to have advanced to a rolling para-oesophageal HH, as evidenced by barium swallow studies and supported by the CT scans. This progression serves as a valuable clinical lesson, highlighting the silent evolution of a benign sliding hernia into a potentially consequential para-oesophageal rolling HH, with implications for gastric volvulus and necessitating surgical correction. 

## Discussion

**Figure 5:**
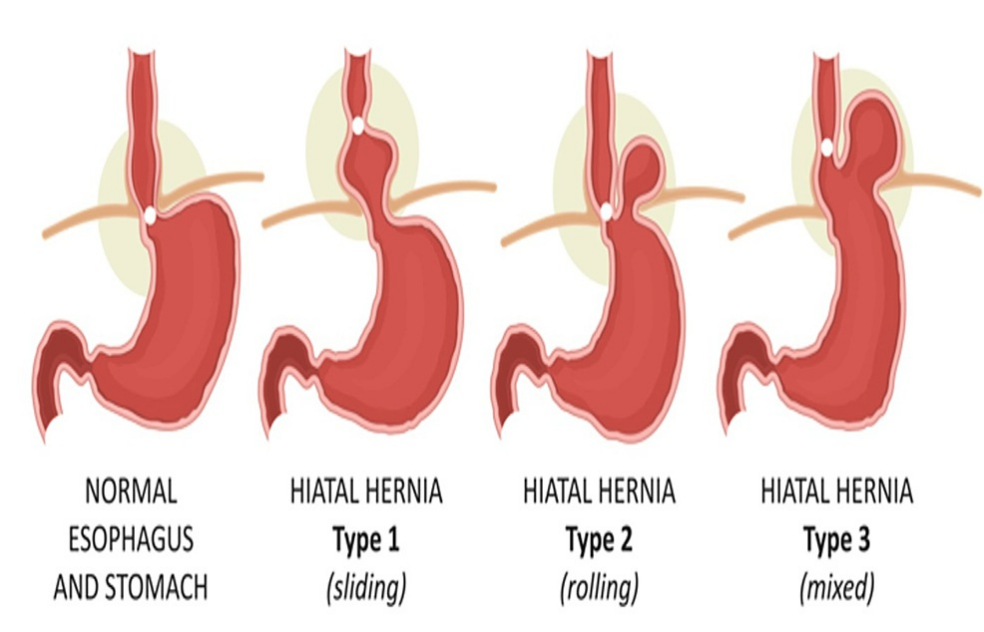
Schematic presentation of the types of oesophageal hiatus hernias. Source: [7] Accessed following purchase and permission. Stock photo and image portfolio by LOGIKA600 Shutterstock. Available at: https://www.shutterstock.com/g/nmfotograf (Accessed: 03 February 2024).

Kumar et al. have reported three types of HH. Type 1 is a sliding HH where the GOJ is displaced above the hiatus; a type 2 or para-oesophageal HH is when the stomach migrates into the mediastinum parallel to the oesophagus (> 2 cm), above the diaphragm. Lastly, a type 3 or mixed HH occurs when both the GOJ and a portion of the stomach have migrated into the mediastinum and 2 cm or more of the fundus is located cephalad to the LOS and oesophagus [5]. A type IV HH has also been reported when the stomach, as well as an additional organ such as the colon, small intestine, or spleen, also herniate into the chest [6]. The different types of HH are shown in Figure 5.

Oesophageal duodenoscopy (OGD), also known as esophagogastroduodenoscopy (EGD), remains the primary investigative tool for assessing the upper gastrointestinal tract. The OGD assists in assessing the competence of the lower end of the oesophagus in two ways. Firstly, by measuring the axial length of the hiatus hernia while retracting the scope represented by the diaphragmatic pinch along the proximal margin of the gastric fold at the GOJ and the GOJ [8]. This axial length of the HH is measured in centimetres on withdrawal of the endoscope [9]. It remains unclear at which length a HH be considered clinically significant since the GOJ or GEJ is not static, still, a cutoff of 2 centimetres or more distal to the squamocolumnar junction (also known as the Z‑line) is considered by most endoscopists to be clinically significant [5].

**Figure 6:**
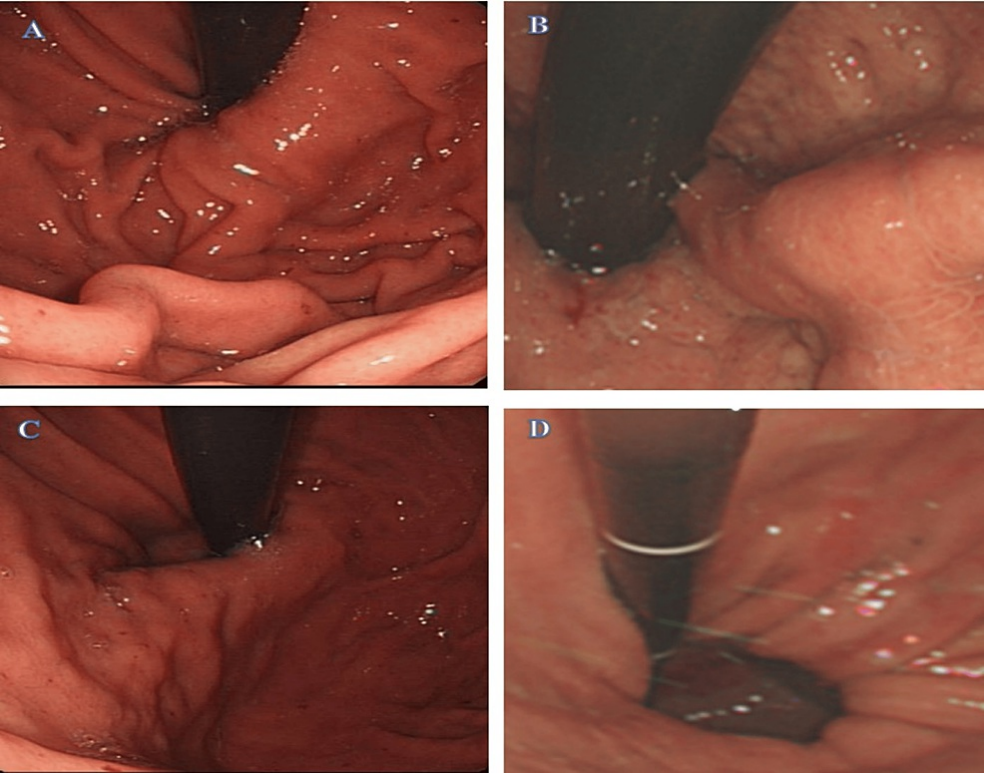
Hill Classification Image Source: Department of Endoscopy. Hammersmith Hospital. Imperial College Healthcare NHS Trust, London, UK [10,15] A: Hill Grade I: a prominent fold of tissue along the lesser curvature next to the endoscope. B: Hill Grade II: the fold is less prominent and there are periods of opening and rapid closing around the endoscope. C: Hill Grade III: the fold is not prominent and the endoscope is not tightly gripped by the tissue D: Hill Grade IV: there is no fold, and the oesophagus lumen is open, often allowing the squamous epithelium to be viewed from below. A hiatal hernia is always present.

The Hill Classification has been reported to be another way to assess the GOJ or GEJ and has been graded to I- IV [10]. The clinical significance of Hill Classification has been reported in various studies and shows a higher Hill grade association with GORD or GERD [11,12], a finding of lesser lower oesophageal sphincter pressure [11], and increased prevalence of hiatus hernia [13]. Lastly, it assists in predicting a poor response to the treatment with PPIs [14]. Hansdotter et al. have shown that Hill Classification, although not superior, still is slightly better than the axial length measurement by endoscopic retraction in the endoscopic assessment of the HH and anti-reflux barrier of GOJ or GEJ [15]. The Hill Classification is illustrated in Figure 6 [10,15].

Several other methods are available to investigate the function of the upper GI. Manometry, for instance, can help rule out primary motility disorders like achalasia, which may mimic symptoms of GORD. Meanwhile, the 24-hour pH monitoring test is considered the gold standard for diagnosing acid reflux. During this procedure, a probe is positioned 5 cm above the GOJ to measure acid exposure levels. The results are then quantified into the DeMeester score, with a score of 14.7 or higher indicating significant gastroesophageal reflux [6]. Additionally, as demonstrated in this case report, an oesophagram utilising barium swallow serves as a valuable tool for assessing a HH, capable of identifying the progression from a sliding to a rolling para-oesophageal HH. Weitzendorfer et al. conducted a study involving 112 patients, comparing barium swallow X-rays with high-resolution manometry studies and OGD. Their findings showed that barium swallow X-ray had the highest rate of HH detection, at 76.8% and is thus the gold standard for detection [16]. Diagnostic endoscopy with the outcome of the HH indirectly has been reported to be associated with GORD [17,18] as in our patient.

The management of an HH involves lifestyle modifications such as weight loss, elevating the head of the bed by eight inches during sleep, and avoiding meals 2-3 hours before bedtime. Additionally, eliminating "trigger" foods like chocolate, alcohol, caffeine, spicy foods, citrus, and carbonated drinks is recommended [19]. The American College of Gastroenterology recommends an eight-week PPI therapy course for symptomatic GORD relief, regardless of the type of PPI used [20]. If the initial response is inadequate, twice-daily PPI therapy is advised [19]. Surgical correction options include a laparoscopic fundoplication, such as Nissen fundoplication (360°) or Toupet fundoplication (270°), for both sliding and para-oesophageal hernias [21]. Patients with pre-existing oesophageal dysmotility may require modified approaches, and obese patients with an HH and GORD may benefit from a sleeve gastrectomy. However, a detailed discussion of surgical techniques is beyond the scope of this case report. 

Here, we present a distinctive case report detailing a patient initially diagnosed with a sliding HH that evolved over time into a rolling HH. This transformation was identified through changes in symptoms and confirmed via comparisons of previous endoscopies, chest X-rays, and CT scans with recent barium swallow studies. Further exploration through additional case reports and research could shed light on the progression of symptoms in patients with HHs, offering valuable insights into the condition's course and management and would assist in avoiding complications like gastric volvulus as reported by Zafar et al. [22]. 

## Conclusions

We advocate for the use of barium swallow studies as a valuable tool in characterising the complexity and clinical course of an HH, even if not considered the gold standard. A prompt diagnosis based on symptom changes, followed by appropriate imaging modalities, can guide effective management. 

Additionally, we recommend that institutions and endoscopy suites mandate the documentation of Hill Classification grades in patients undergoing OGD with retroflexion. This standardised approach would enhance diagnostic accuracy and inform treatment decisions. 

Lastly, we emphasise the importance of utilising not only endoscopy and manometry but also simple imaging modalities such as chest X-rays, CT scans, and barium swallow studies. Recognising symptom changes over time and promptly investigating them is crucial for accurate diagnosis and management. This case emphasises the significance of identifying evolving symptoms suggestive of different forms of hernia along a continuum, highlighting the need for vigilant clinical assessment and investigation. 
